# Interleukin-4 protects retinal ganglion cells and promotes axon regeneration

**DOI:** 10.1186/s12964-024-01604-y

**Published:** 2024-04-22

**Authors:** Zhaoyang Zuo, Bin Fan, Ziyuan Zhang, Yang Liang, Jing Chi, Guangyu Li

**Affiliations:** Department of Ophthalmology, The Second Norman Bethune Hospital of Jilin University, 130041 Changchun, China

**Keywords:** Retinal ganglion cells, Optic nerve crush, Nerve excitotoxicity, Axon regeneration

## Abstract

**Background:**

The preservation of retinal ganglion cells (RGCs) and the facilitation of axon regeneration are crucial considerations in the management of various vision-threatening disorders. Therefore, we investigate the efficacy of interleukin-4 (IL-4), a potential therapeutic agent, in promoting neuroprotection and axon regeneration of retinal ganglion cells (RGCs) as identified through whole transcriptome sequencing in an in vitro axon growth model.

**Methods:**

A low concentration of staurosporine (STS) was employed to induce in vitro axon growth. Whole transcriptome sequencing was utilized to identify key target factors involved in the molecular mechanism underlying axon growth. The efficacy of recombinant IL-4 protein on promoting RGC axon growth was validated through in vitro experiments. The protective effect of recombinant IL-4 protein on somas of RGCs was assessed using RBPMS-specific immunofluorescent staining in mouse models with optic nerve crush (ONC) and N-methyl-D-aspartic acid (NMDA) injury. The protective effect on RGC axons was evaluated by anterograde labeling of cholera toxin subunit B (CTB), while the promotion of RGC axon regeneration was assessed through both anterograde labeling of CTB and immunofluorescent staining for growth associated protein-43 (GAP43).

**Results:**

Whole-transcriptome sequencing of staurosporine-treated 661 W cells revealed a significant upregulation in intracellular IL-4 transcription levels during the process of axon regeneration. In vitro experiments demonstrated that recombinant IL-4 protein effectively stimulated axon outgrowth. Subsequent immunostaining with RBPMS revealed a significantly higher survival rate of RGCs in the rIL-4 group compared to the vehicle group in both NMDA and ONC injury models. Axonal tracing with CTB confirmed that recombinant IL-4 protein preserved long-distance projection of RGC axons, and there was a notably higher number of surviving axons in the rIL-4 group compared to the vehicle group following NMDA-induced injury. Moreover, intravitreal delivery of recombinant IL-4 protein substantially facilitated RGC axon regeneration after ONC injury.

**Conclusion:**

The recombinant IL-4 protein exhibits the potential to enhance the survival rate of RGCs, protect RGC axons against NMDA-induced injury, and facilitate axon regeneration following ONC. This study provides an experimental foundation for further investigation and development of therapeutic agents aimed at protecting the optic nerve and promoting axon regeneration.

**Supplementary Information:**

The online version contains supplementary material available at 10.1186/s12964-024-01604-y.

## Background

Retinal ganglion cell (RGC) death and axon loss constitute the primary pathological characteristics of various blinding disorders, including glaucoma and traumatic optic neuropathy. Consequently, protecting RGCs and facilitating the regeneration of RGC axons emerge as pivotal concerns in the therapeutic management of these diseases.

Staurosporine (STS) is a broad-spectrum inhibitor of protein kinase C (PKC). It has been demonstrated that high concentrations of staurosporine can induce apoptosis in nerve cells [1]. Conversely, at nanomolar levels, staurosporine significantly protects retinal ganglion cells in mice with optic nerve injury [2]. In vitro studies have revealed that low concentrations of staurosporine effectively promote the growth and development of neurons or neuron-like cells such as HN33 and PC12, promoting the formation of axon-like structures [3, 4]. Furthermore, Kim et al. reported that treatment with 50 nM staurosporine for 12 h induces differentiation in chicken embryonic retinal ganglion cells [5]. These findings collectively suggest that low concentrations of staurosporine activate a shared molecular mechanism to enhance axon outgrowth across various neuronal types. This knowledge may serve as a foundation for identifying crucial targets involved in retinal ganglion cell protection and axon regeneration in vitro.

Furthermore, extracellular factors, especially cytokines, also play a crucial role in the process of axon regeneration. Cytokines are multifunctional and multi-effect proteins that not only participate in immune responses but also participate in pathological processes after central nervous system (CNS) injury. Numerous studies have demonstrated that local T cells are stimulated, activated and secrete different cytokines after neuron injury in CNS of mice and rats [6]. Additionally, Fischer et al. revealed that continuous expression of hyper-IL-6 directly targeting the gp130 receptor can activate JAK/STAT3 and PI3K/AKT/mTOR signaling pathways with greater effect on promoting axon regeneration of retinal ganglion cells than other known treatments (such as *PTEN* knockout) [7]. Bethea et al. reported that IL-10 inhibited TNF-α production and promoted axon regeneration of dorsal root ganglion cells after spinal cord injury in rats [8]. Moreover, Lindborg et al. showed that inhibition of IL-22 could increase the levels of transcription factors and cytokines that play an important role in optic nerve regeneration signals, and significantly promote the axon regeneration of retinal ganglion cells in mice [9]. Consequently, cytokines assume a critical role in neuroprotection and axon regeneration.

N-methyl-D-aspartic acid receptor (NMDAR) is a subtype of glutamate receptor. Overactivation of NMDARs can trigger calcium influx and excessive production of nitric oxide, leading to apoptosis and subsequent retinal ganglion cell death. N-methyl-D-aspartic acid (NMDA) primarily induces RGC death by nerve excitotoxic damage, followed by anterograde degeneration of RGC axons [10]. However, the optic nerve crush (ONC) model damages the axon structure of the optic nerve through mechanical force, resulting in increased calcium influx, inflammatory response, oxidative stress damage, and ultimately retrograde RGC death [11]. Due to distinct injuries, the processes and molecular mechanisms underlying RGC death and axon degeneration exhibit partially different. In addition, previous studies have shown that different types of RGCs display varying susceptibilities to ONC or NMDA-induced injury. BD-RGC represents the most vulnerable RGC subtype to ONC injury, whereas W3-RGC exhibits heightened sensitivity to the NMDA damage [12]. Utilizing both injury models will facilitate the identification of shared molecular mechanisms and potential therapeutic targets for RGC death caused by diverse injuries.

The present study established an in vitro model of axon growth using staurosporine (50 nM) in 661 W cells. Subsequently, scRNA-seq was employed to screen for the candidate factor IL-4, which plays a crucial role in promoting axon growth. Furthermore, we investigated the protective effect of recombinant IL-4 protein (rIL-4) on retinal ganglion cells (RGCs), as well as its potential to enhance axon regeneration through NMDA and ONC injury models in vivo. These findings lay a solid experimental foundation for the development of novel therapeutic strategies targeting optic nerve protection and regeneration.

## Materials and methods

### Animals

Five- to six-week-old male C57BL/6J mice (WT) weighing 20 to 25 g were purchased from the Animal Research Center of Jilin University (Changchun, Jilin, China). All mice purchased were housed in a central animal care facility with room temperature maintained at 21 ℃ − 23 ℃ and a 12-h light/dark cycle maintained, and mice had free access to food and water. All animal experiments were reviewed and approved by the Ethics Committee of Jilin University and complied with the ARVO Declaration on Animal Research.

### Cell culture

661 W cells were gifted to Dr. Muayyad Al-Ubaidi (University of Oklahoma Health Sciences Center, USA). 661 W cells were cultured at 37 °C in a 5% CO_2_ cell incubator in Dulbecco’s modified Eagle medium (HyClone, Beijing, China) supplemented with 10% fetal bovine serum and 1% penicillin/streptomycin. The cells were passaged every 2–3 days using trypsin-EDTA.

### Cell differentiation with STS and rIL-4

The culture was continued for 24 h after the addition of STS to a final concentration of 50 nM or recombinant murine IL-4 protein to a final concentration of 1 µg/mL in each well of a tissue culture six-well plate inoculated with 661 W cells.

### Immunofluorescence staining of 661 W cells

The cells were fixed in 4% paraformaldehyde, permeabilized with 0.5% Triton X-100 for 20 min, blocked with 5% BSA for 1 h, and then incubated with the corresponding primary antibody (mouse anti-βIII-Tubulin, 1:100; rabbit anti-NeuN, 1:400, 4 ℃ overnight). The cells were washed three times with PBS (5 min) and incubated with the corresponding secondary antibodies (DyLight 488, anti-mouse IgG, 1:400; Cy3, anti-rabbit IgG, 1:400, 2 h at room temperature in the dark). After further staining of nuclei with DAPI, samples were placed under a fluorescence microscope (Olympus, Japan), observed and photographed. Neurons with positive immunofluorescence staining were traced and analyzed using the SNT plug-in of ImageJ.

### Immunoblotting

Cells or retinal tissue were ultrasonically disrupted in 1% protease inhibitor (PMSF) and RIPA protein lysis buffer (Beyotime, Shanghai, China). The protein concentration was measured using a BCA kit (Beyotime, Shanghai, China). Cell lysates were dissolved in an equal volume (20 µg) of sample buffer and boiled at 100 ℃ for 5 min, followed by electrophoresis on an 8–12% SDS-polyacrylamide gel. Next, the proteins were transferred to PVDF membranes. PVDF membranes were blocked with 5% skim milk. The corresponding primary antibodies were then added for incubation (4 ℃, overnight) (anti-NeuN antibody, 1:2000, anti-βIII-tubulin antibody, 1:2000, Beyotime, Shanghai, China). After washing three times (5 min), the corresponding horseradish peroxidase conjugated secondary antibody (anti-rabbit IgG, HRP conjugated/anti-mouse IgG, HRP conjugated, 1:8000, Signalway, Pearland, TX, USA) was added and incubated for 1 h. The target protein blots were visualized using the enhanced chemiluminescence method and photographed with a charge coupled device (CCD) camera (Tanon, Shanghai, China), and the gray value of the protein bands was analyzed by ImageJ software.

### mRNA sequencing

The sequencing samples were 50 nM STS-treated 661 W cells and untreated 661 W cell samples in triplicate for each group. Total RNA in cells was isolated using TRIzol (Invitrogen, Carlsbad, CA, USA). The quality was then quantified and assessed using a NanoDrop and Agilent 2100 bioanalyzer (Thermo Fisher Scientific, MA, USA). After mRNA purification using oligonucleotide (dT)-attached magnetic beads, cDNA fragments were generated by reverse transcription with random hexamer primers and amplified by polymerase chain reaction (PCR). After the reaction was purified with Ampure XP Beads, it was dissolved in ethidium bromide solution. Double-stranded PCR products were validated using an Agilent Technologies 2100 bioanalyzer and then heated, denatured, and cycled through splinted oligonucleotide sequences to obtain the final library. The final library was amplified with phi29 to form DNA nanospheres (DNBS) containing more than 300 copies of a single molecule. DNB was loaded into the regular nanoarray, and 50-bp single-end sequencing reads were generated on the BGIseq500 platform (BGI-Shenzhen, China). Clean reads were obtained and stored in FASTQ format after removing reads with adaptor sequences, low-quality base ratios greater than 20% (base quality less than or equal to 5) or unknown bases (“N” base) ratio greater than 5% using SOAPnuke (v1.5.2). The clean reads were mapped to the reference genome using HISAT2 (version: LU_Bosgru_v3.0). The clean reads were aligned to the reference coding gene set using Bowtie2 (v2.2.5), and then the expression of the gene was calculated using RSEM (v1.2.12). Finally, using the R package DESeq2b (v1.4.5) differential expression analysis, data were reported for log2 (STS_samples/Vehicle_samples). The threshold was a|log2FoldChange| ≥ 1 or q ≤ 0.05.

### NMDA model, optic nerve crush model and drug administration

After anesthesia with 1% pentobarbital sodium, the pupil of the mouse was dilated with a mixture of 0.5% tropicamide and 0.5% phenylephrine hydrochloride eye drops (Santen, Suzhou, China). Scleral puncturing was performed under an operating microscope using a 32G needle at approximately 1–1.5 mm posterior to the limbus, tilted downward. A 10 µl Hamilton syringe (Hamilton Company, Reno, NV) was attached to a 33 G needle and inserted into the vitreous cavity through the scleral tunnel while avoiding damaging the lens. A total of 1.5 µl of drug was injected into the vitreous cavity each time.

To establish the ONC injury model, adult mice were anesthetized with 1% pentobarbital sodium and placed under the operating microscope. Oxybuprocaine hydrochloride ophthalmic solution (0.5%; Santen, Suzhou, China) was used for topical anesthesia. Following the blunt separation of the conjunctival sac using two toothed forceps around the lateral canthus, the optic nerve of the left eye was carefully exposed intraorbitally and crushed with self-closing Dumont forceps #5 (Beijing, China) 1 mm behind the globe for 15 s without damaging the ophthalmic artery. Then, rIL-4 (0.1 µg/µl) was injected into the left eye, and vehicle was injected into the fellow eye as a control on days 0 and 3 for the 7-day group. The mice in the 14-day group were treated on days 0, 3, and 7.

To establish the NMDA injury model, NMDA (20 mM) and rIL-4 (0.1 µg/µl) were injected into the left eye, and NMDA and vehicle were injected into the fellow eye on day 0. Then, rIL-4 was injected into the left eye and vehicle was injected into the fellow eye on day 3 for the 7-day group, and on days 3 and 7 for the 14-day group. Cholera toxin B subunit (CTB) was injected into the eyes of the mice 3 days before euthanasia.

### Histology

After the mice were anesthetized with 1% sodium pentobarbital, the whole body was perfused with normal saline and 4% PFA for prefixation. Next, the eyeball, optic nerve, optic chiasm, and brain were dissected and isolated. Tissues were fixed in 4% PFA for 24 h followed by dehydration in 30% sucrose for 24 h.

Retinas were isolated from mouse eyeballs, and retinal flat mounts were made. The flat mounts were permeabilized in 0.5% Triton X-100 for 30 min at room temperature and blocked in 10% normal goat serum, 1% BSA and 0.1% Triton X-100 in PBS for 1 h. Next, the flat mounts were incubated overnight in the corresponding primary antibodies (rabbit anti-RBPMS, 1:200). After washing three times (10 min) with PBS, the flat mounts were incubated with the corresponding secondary antibody (DyLight 488, anti-rabbit IgG, 1:400) for 2 h (room temperature) and washed three times (10 min) with PBS. The flat mounts were mounted before being observed under a microscope and photographed. Images of the whole retinal flat mount were stitched from several low-magnification images using Adobe Photoshop software. Twelve images (0.35 × 0.45 mm^2^ each) were taken for each retina at high magnification using a fluorescence microscope (Olympus, Japan). The number of RGCs in each image was counted with the StarDist 2D plug-in in ImageJ software.

To prepare frozen sections of retinas and optic nerves, fixed eyeballs, with anterior segments and vitreous removed, or optic nerves were sectioned (10 μm for axial sections of retinas and optic nerves and 4 μm for cross-sectional sections of optic nerves). For CTB-labeled samples, frozen sections were mounted and observed under a fluorescence microscope (Olympus, Japan).

Frozen sections that needed immunofluorescence staining were washed with PBS and blocked with 10% normal goat serum, 1% BSA, and 0.1% Triton X-100 in PBS for 1 h. Next, the frozen sections were incubated (4 ℃, overnight) with the corresponding primary antibodies (rabbit anti-GAP43, 1:200; mouse anti-β-III Tubulin, 1:100). The sections were washed (10 min) three times with PBS and incubated with the corresponding secondary antibody for 2 h (Cy3, anti-rabbit IgG, 1:400; DyLight 488, anti-mouse IgG, 1:400, room temperature). The slides were washed three times (5 min) with PBS at room temperature, mounted and observed under a fluorescence microscope (Olympus, Japan). For each group of ONC models, the regeneration of axons at a prespecified distance from the injury site was calculated by counting the number of fluorescent positive axons at 500 μm from the injury site and measuring the width of the optic nerve at that location (*r* is the radius of the optic nerve, *t* is the section thickness). The total number of regenerated axons with lengths greater than or equal to 500 μm was calculated. The formula is Σ_ad_ = π*r*^2^ × (average number of axons/width (mm))/*t*.

After immunofluorescence staining of cross-sectional sections of the optic nerve for β-III tubulin, the number of β-III tubulin-positive axons 1 cm from the injury site was counted using the Stardist plugin in ImageJ. Five 20 μm× 20 μm square areas were taken from each cross-sectional section of optic nerve for analysis, counting, and calculation of density. Then, the mean value of nerve density was calculated for each group.

For CTB labeling in the lateral geniculate body and superior colliculus, mouse brains were collected 3 days after intravitreal injection of CTB. Frozen Sect. (15 μm for LGN and SC) were obtained coronally at the LGN and SC. Thereafter, the frozen sections were mounted with mounting medium. The frozen sections were placed under a fluorescence microscope (Olympus, Japan) for observation and photography.

### Flash electroretinogram (ERG)

Flash ERG was performed with the RetiMINER IV system (IRC Medical Equipment, Chongqing, China). For the NMDA injury model, the mice in each group were adapted in darkness overnight before the beginning of experiment. All procedures were performed under a dim red light to maintain scoptic vision. After anesthetizing with tribromoethanol (350 mg/kg), atropine sulfate eye gel was used to dilate pupils. Subsequently, thread electrodes were placed across the center of bilateral cornea. A subcutaneous electrode was inserted subcutaneously between bilateral eyes. A ground electrode was placed at the end of the mouse’s tail with conductive paste. Bilateral ERG recording was performed from both eyes simultaneously, with each item repeated at least twice. The reliability of the recorded waveforms was confirmed by the coincidence among waveforms. Scotopic ERGs were recorded at stimulus intensities levels of 0.001, 0.01, 0.1, 1.0, 3.0, and 10.0 cd·s/m^2^. Oscillatory potentials (OPs) were recorded using the white flashes of 3.0 cd·s/m^2^ scotopic responses via bandpass filtering between 50 and 170 Hz. The positive scotopic threshold responses (pSTRs) were measured from the baseline to the positive peak of the waveform at a flash intensity of 0.001 cd·s/m^2^. After light adaptation for 15 min, the photopic negative responses (PhNRs) were measured from the baseline to the trough of the negative response following the positive b-wave at a flash intensity of 10.0 cd·s/m^2^.

### Chemicals and reagents

The chemicals and reagents used in this study are listed in Table 1.

**Table 1:** Chemicals and reagents used in the study

Chemicals	Source	Identifier	Dosage
Staurosporine	Abmole (Beijing, China)	Cat# M2066	
N-methyl-D-aspartic Acid (NMDA)	Abmole	Cat# M2884	
4’,6-diamidino-2-phenylindole (DAPI)	Solarbio (Beijing, China)	Cat# C0065	
Antifading mounting medium	Solarbio	Cat# S2100	
Cholera Toxin Subunit B (CTB) conjugated Alexa Fluor 488	Thermo Fisher Scientific (MA, USA)	Cat# C34775	
recombinant murine IL-4 protein	PeproTech (Rocky Hill, NJ, USA)	Cat# 214 − 14	
**Reagents for cell histology**	**Source**	**Identifier**	**Dosage**
mouse anti-βIII-Tubulin	Beyotime (Shanghai, China)	Cat# AT809	1:100
rabbit anti-NeuN	Abcam (Cambridge, UK)	Cat# ab177487	1:400
DyLight 488, anti-mouse IgG	Abbkine (Shanghai, China)	Cat# A23210	1:400
Cy3, anti-rabbit IgG	Abbkine	Cat# A22220	1:400
**Reagents for Western blot**	**Source**	**Identifier**	**Dosage**
mouse anti-β-III Tubulin	Beyotime	Cat# AT809	1:2000
rabbit anti-NeuN	Abcam	Cat# ab177487	1:5000
mouse anti-β-actin	Signalway (Pearland, TX, USA)	Cat# 21,800	1:8000
anti-rabbit IgG, HRP conjugated	Signalway	Cat# L3012	1:8000
anti-mouse IgG, HRP conjugated	Signalway	Cat# L3032	1:8000
**Reagents for tissue histology**	**Source**	**Identifier**	**Dosage**
rabbit anti-RBPMS	OmnimAbs (California, USA)	Cat# OM165217	1:200
rabbit anti-GAP43	Beyotime	Cat# AF0153	1:200
mouse anti-β-III Tubulin	Beyotime	Cat# AT809	1:100
rabbit anti-IL4R	Affinity Biosciences	Cat# DF8567	1:200
DyLight 488, anti-rabbit IgG	Abbkine	Cat# A23220	1:400
DyLight 488, anti-mouse IgG	Abbkine	Cat# A23210	1:400
Cy3, anti-rabbit IgG	Abbkine	Cat# A22220	1:400

### Statistical analysis

All experiments were repeated at least three times, and values are presented as the mean ± standard error of the mean (mean ± SEM). An unpaired two-tailed Student’s *t* test was used for comparisons between the two groups. One-way analysis of variance (ANOVA) was used to assess mean differences among multiple groups, followed by Bonferroni post hoc correction or Dunn’s nonparametric Kruskal‒Wallis test for multiple comparisons. ImageJ software was used to measure the gray value of the Western blot results. GraphPad Prism version 9.0.0 for Windows (GraphPad Software, San Diego, CA, USA) was used for data processing, analysis, and plotting. A value of *p* < 0.05 was considered statistically significant.

## Results

### STS induces differentiation and axon outgrowth of 661 W cells

**Fig. 1:**
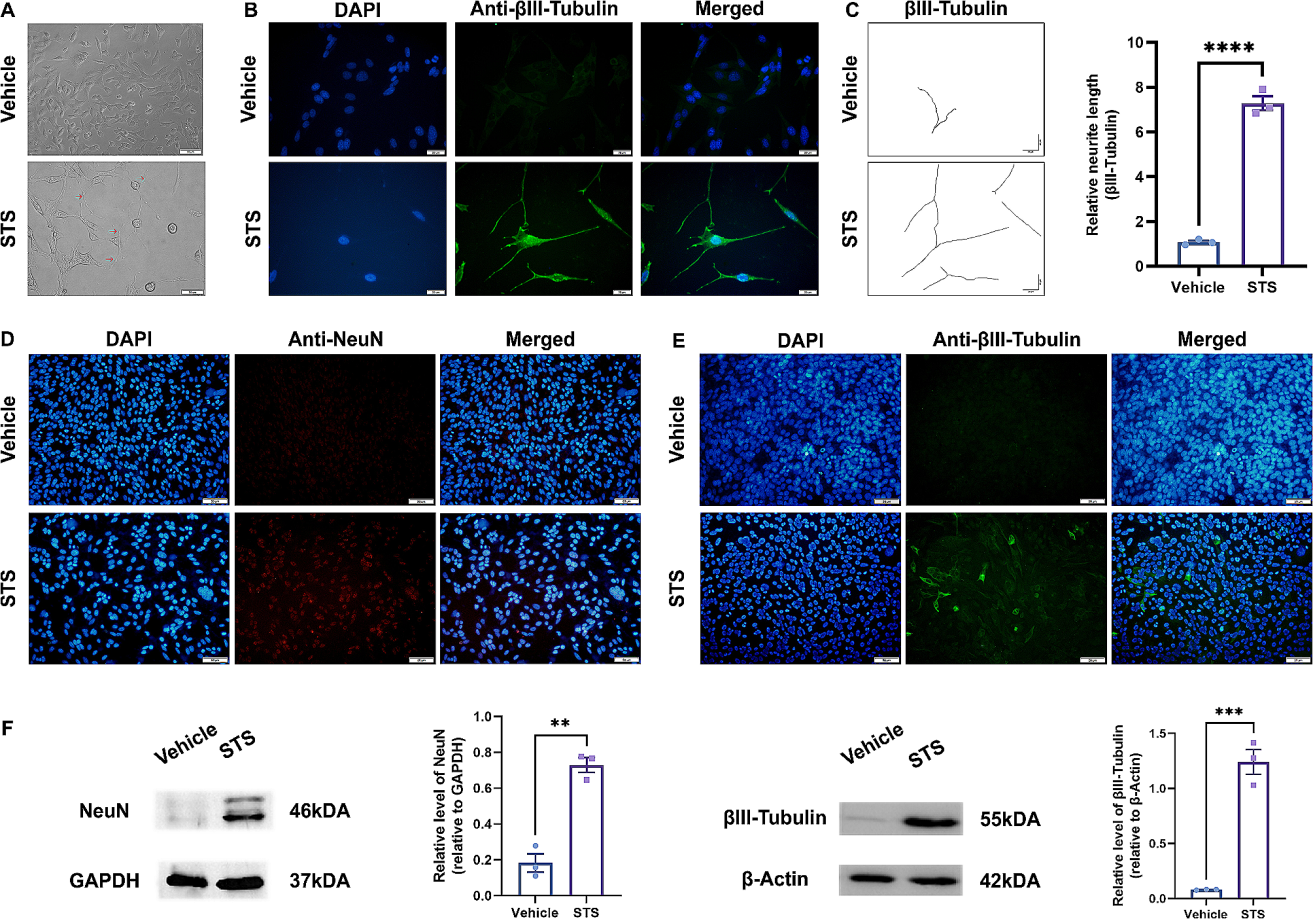
Induction of axon outgrowth in 661 W cells by STS. **A**: Light microscopy showed that 661 W cells treated with 50 nM STS for 6 h extended long axon-like structures (indicated by red arrows). **B**: The extended axon-like structures of 661 W cells treated with 50 nM STS showed positive immunofluorescence staining for βIII-tubulin. C: Neurite tracing showed that the extended neurites of 661 W cells treated with 50 nM STS were longer than those treated with vehicle. D, E: Higher fluorescence intensity of NeuN (red) and βIII-tubulin (green) was observed in 661 W cells treated with STS. F: Western blot results showed that the protein levels of NeuN and βIII-tubulin were higher in 661 W cells treated with STS. n = 3 per group, ** indicates *p* < 0.01, *** indicates *p* < 0.005, **** indicates *p* < 0.001

As depicted in Fig. 1A, treatment of 661 W cells with a concentration of 50 nM STS for a duration of 6 h significantly facilitated the growth of axon-like structures. Immunofluorescence staining further confirmed the strong positivity of β-III tubulin in these developing neurites (Fig. 1B). Additionally, compared to the vehicle group, the STS-treated group exhibited a substantial increase in neurite extension length (Fig. 1C). Furthermore, immunostaining and Western blot results demonstrated significant upregulation of neuronal markers such as NeuN and β-III tubulin in STS-treated 661 W cells, indicating that STS effectively induced differentiation into mature neurons (Fig. 1D-F).

**Fig. 2:**
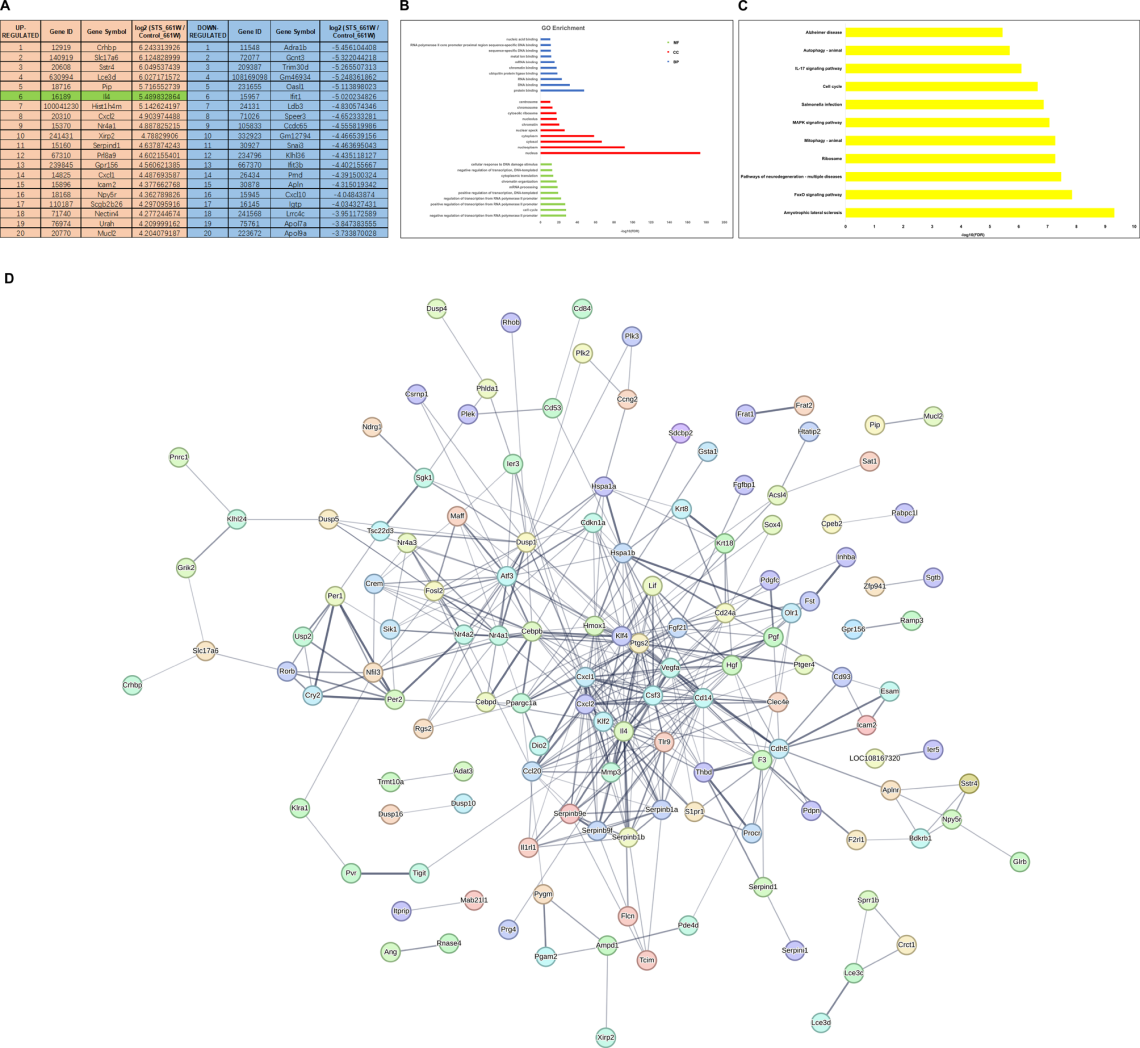
Enrichment analysis of upregulated mRNAs in STS-treated 661 W cells. **A**: Differentially expressed mRNAs in 661 W cells treated with STS compared with 661 W cells treated with vehicle were ranked according to the changes in transcriptional levels. The top twenty upregulated or downregulated mRNAs are listed. IL-4 was located at the 6th position of all upregulated mRNAs. **B**: Enrichment analysis of biological process, cellular component and molecular function of upregulated mRNAs. **C**: KEGG pathway enrichment analysis results. **D**: Protein‒protein interaction analysis of the top 200 upregulated mRNAs. IL-4 was the central node

To elucidate the molecular mechanism underlying STS-induced axon outgrowth, we conducted whole transcriptome RNA sequencing on 661 W cells treated with STS. Based on the differential expression of genes in response to STS treatment, upregulated and downregulated genes were ranked according to their transcriptional changes. Notably, the transcription level of IL-4 was significantly increased (Fig. 2A). Subsequently, we employed the Gene Ontology (GO) database for enrichment and analysis of biological processes, cellular components, molecular functions. We also KEGG pathway analysis with upregulated genes following STS treatment. Our KEGG pathway analysis revealed that differentially transcribed genes primarily participated in inflammation-related signaling pathways including the IL-17 signaling pathway, MAPK pathway, and autophagy pathway (Fig. 2B and C). Furthermore, protein-protein interaction analysis demonstrated that IL-4 occupied a central position within the network of upregulated signals in 661 W cells (Fig. 2D). Collectively, these findings suggest that IL-4 may represent a promising molecular target through which STS promotes axon growth.

### Recombinant IL-4 protein promotes the differentiation and axon outgrowth of 661 W cells

**Fig. 3:**
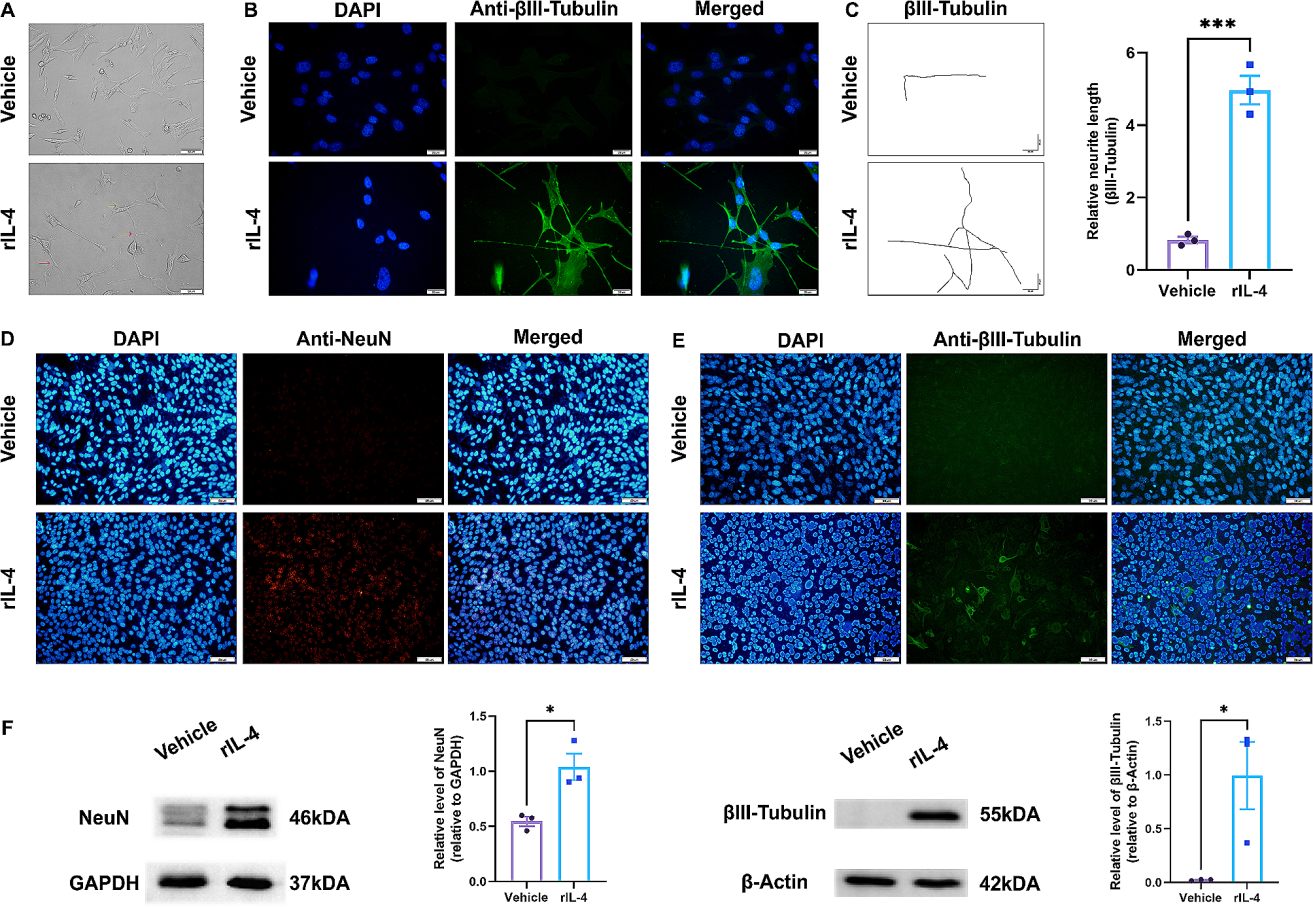
Induction of axon outgrowth by recombinant IL-4 protein in 661 W cells. **A**: Light microscopy image of 661 W cells treated with vehicle and 1 µg/mL rIL-4 for 24 h. Axon-like structures (indicated by red arrows) extended from 661 W cells in the rIL-4 group. **B**: Immunofluorescence staining of βIII-tubulin showed that the axon-like structure extended from 661 W cells in the rIL-4 group was βIII-tubulin positive. **C**: Neurite tracing showed that the neurite extension length of the rIL-4 group was significantly longer than that of the vehicle group at the same treatment time. **D**, **E**, **F**: The fluorescence intensity of NeuN (red) and βIII-tubulin (green) in the rIL-4 group was higher than that in the vehicle group. Western blot results showed that the protein levels of βIII-tubulin and NeuN in the rIL-4 group were higher than those in the vehicle group. n = 3 per group, * indicates *p* < 0.05, *** indicates *p* < 0.005

Subsequently, we investigated the favorable impact of recombinant IL-4 protein on axon growth in 661 W cells. Following a 24-hour treatment with 1 µg/mL recombinant IL-4 protein, noticeable growth of axon-like structures was observed in the treated 661 W cells (Fig. 3A). These developing structures exhibited positive staining for βIII-tubulin (Fig. 3B) and displayed longer extensions compared to those in the vehicle group (Fig. 3C). Furthermore, treatment with recombinant IL-4 protein induced specific neuronal characteristics in the 661 W cells, as evidenced by significantly increased expression levels of NeuN and βIII-tubulin (Fig. 3D, E). Western blot analysis also confirmed a significant upregulation of NeuN and βIII-tubulin expression in the recombinant IL-4-treated 661 W cells (Fig. 3F). In conclusion, our findings demonstrate that recombinant IL-4 protein effectively promotes axon growth in 661 W cells similar to the effects exerted by STS.

### Recombinant IL-4 protein protected RGSs from NMDA damage

**Fig. 4:**
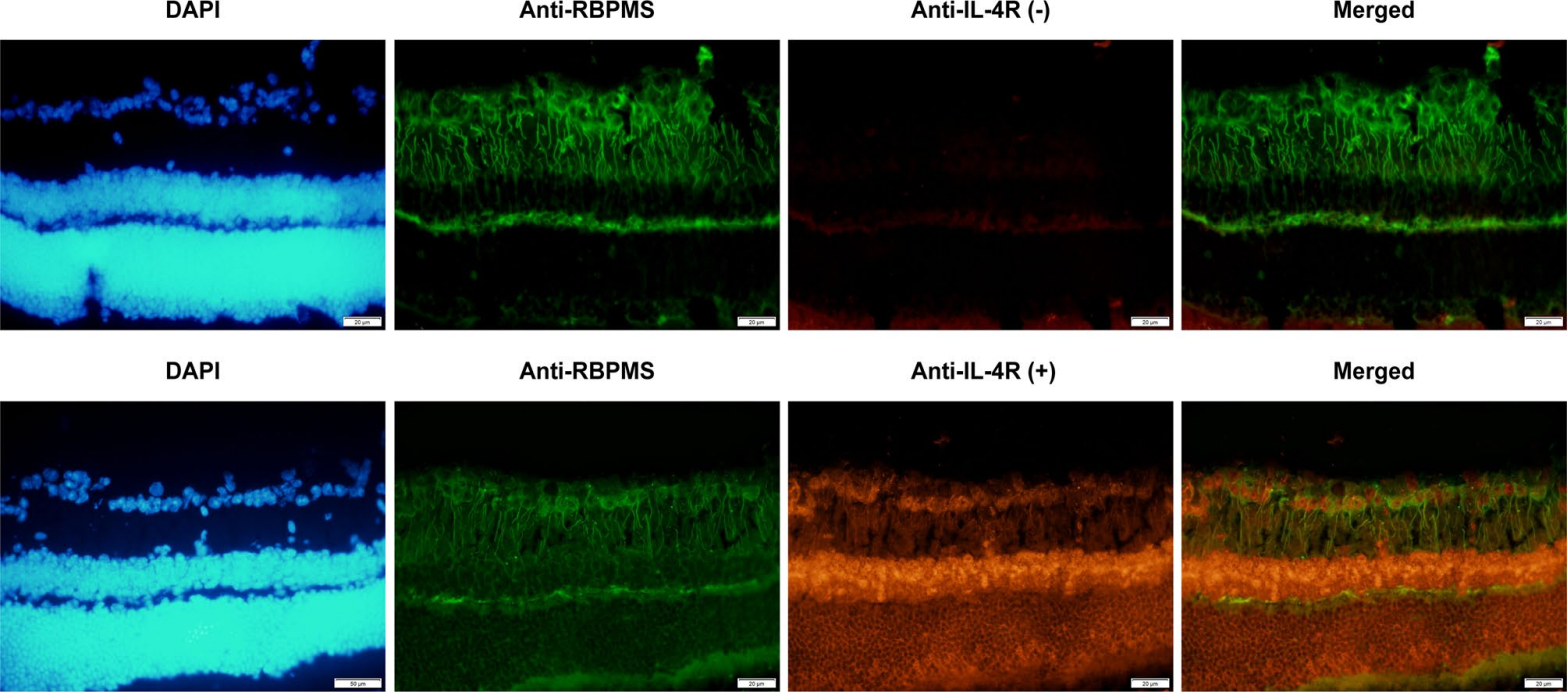
IL-4 receptors expressed on mouse RGCs. RGCs were labeled with RBPMS (green), and IL-4 receptors were labeled with an IL-4R antibody (red). First row: control group without IL-4 receptor antibody incubation; Row 2: IL-4 receptor was expressed in the RGC layer. The red fluorescence labeled IL-4 receptor coincided with the green fluorescence labeled RGC, suggesting that IL-4 receptors were expressed on RGCs

To further investigate the effects of the recombinant IL-4 protein on RGCs, two animal models of RGC axon injury were established. Initially, we examined the expression of IL-4 receptors in mouse retinal RGCs. The biological activity of exogenous IL-4 relies on its specific interaction with IL-4 receptors present on the cell surface. Immunofluorescence staining results demonstrated that interleukin-4 receptor (IL-4R) was expressed in the inner layer neurons of the mouse retina. The subsequent double immunofluorescence staining confirmed the IL-4R was in the RGC layer (Fig. 4). These findings suggested that the recombinant IL-4 protein may exert its biological effects through receptor-mediated signaling pathways in retinal ganglion cells.

**Fig. 5:**
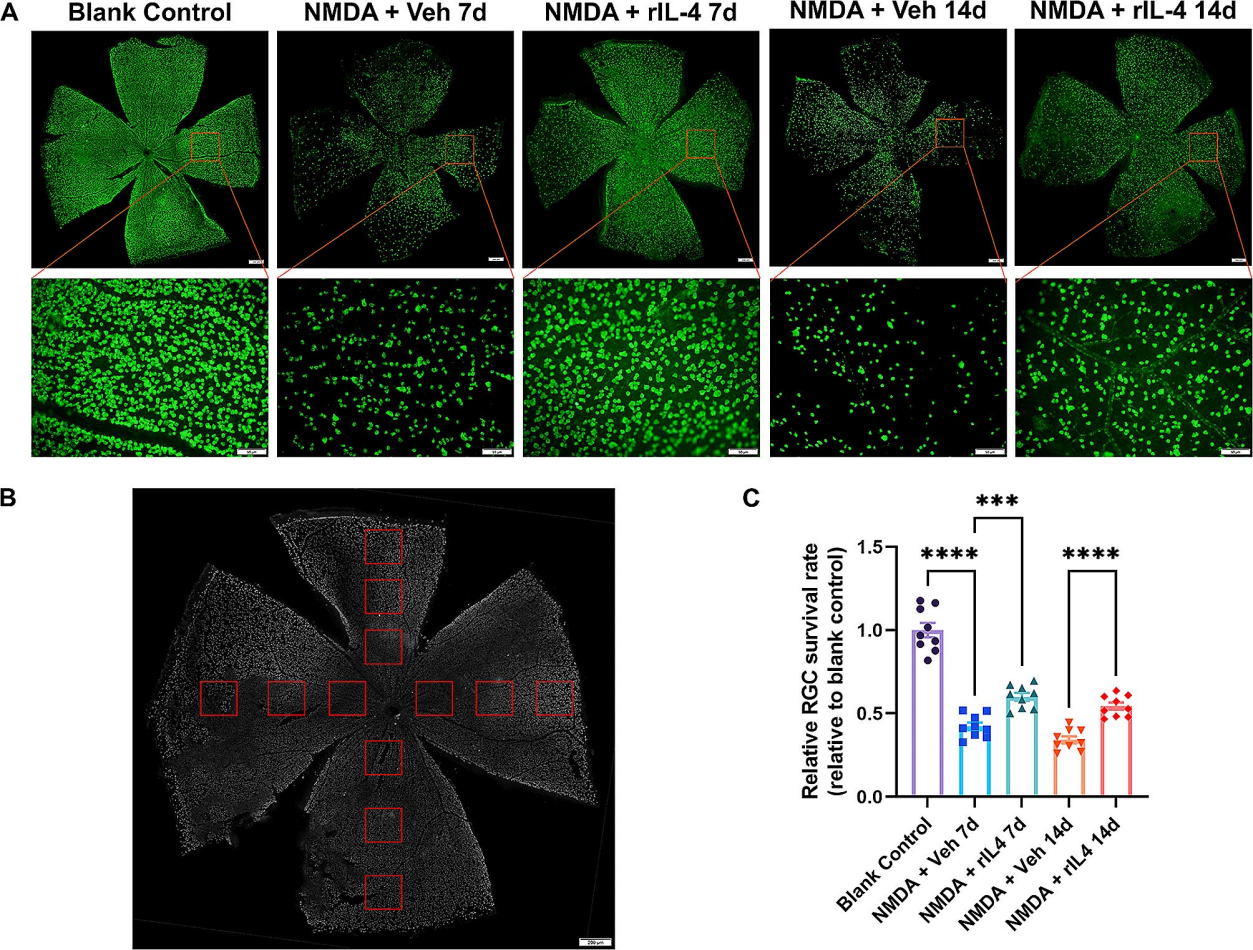
Recombinant IL-4 protein reduced RGC cell death caused by NMDA injury. **A**: Immunofluorescence staining of retinal ganglion cells in the negative control group, vehicle group and rIL-4 group on day 7 and day 14 showed that the density of RGCs in the rIL-4 group was higher than that in the vehicle group at the same treatment time. **B**: Schematic diagram of the RGC counting method. A rectangle (red) indicates a high-power field of 0.38 mm×0.285 mm. The number of RGCs in each high-power field was counted, and the mean RGC number in a high-power field was calculated. The proportion of the mean RGC number of the vehicle group or rIL-4 group relative to the mean RGC number of the negative control group was calculated as the relative RGC survival rate. **C**: The relative RGC survival rate of each group. The relative RGC survival rate of the IL-4 group was greater than that of the vehicle group with the same treatment time. n = 9 eyes per group, *** indicates *p* < 0.005, **** indicates *p* < 0.001

Afterward, we examined the protective efficacy of intravitreal administration of recombinant IL-4 protein (20 mM) against NMDA-induced RGC death. As depicted, treatment with recombinant IL-4 protein significantly conferred protection to RGCs on days 7 and 14 post-NMDA injury (Fig. 5A, B). The rIL-4 group exhibited a substantially higher number of surviving RGCs compared to the vehicle group (vehicle group vs. rIL-4 group, day 7: 576 ± 41 vs. 724 ± 51, *p* < 0.05; Day 14: 457 ± 53 vs. 612 ± 72, *p* < 0.05). On day 14, the average number of surviving RGCs in the rIL-4 group was approximately 1.3-fold greater than that in the vehicle group. These findings strongly indicate that recombinant IL-4 protein confers robust neuroprotection against NMDA-induced injury to RGCs.

### The recombinant IL-4 protein protected RGC axons from NMDA injury

**Fig. 6:**
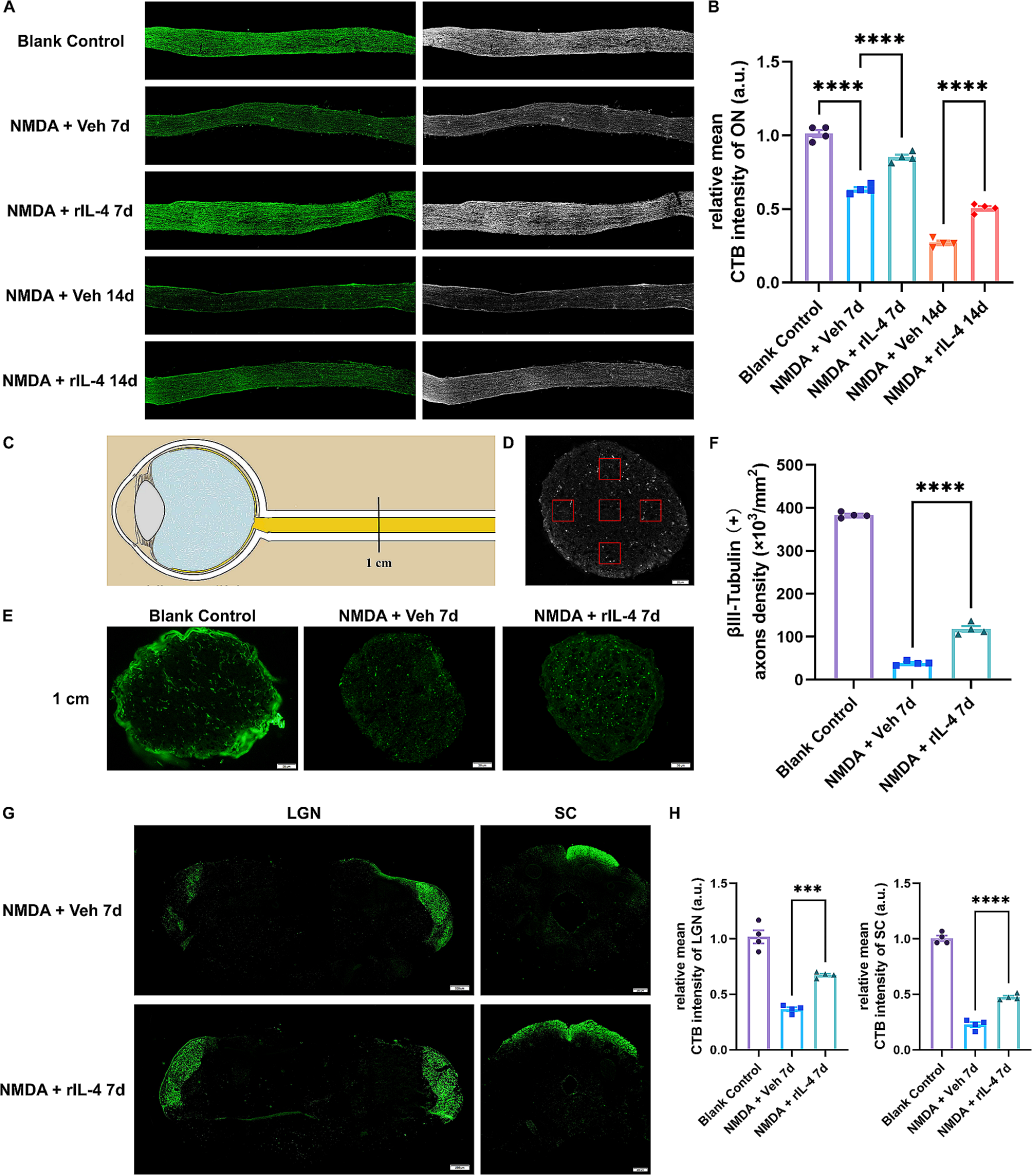
Recombinant IL-4 protein attenuated NMDA-induced axon degeneration and maintained long-range axon projections in RGCs. **A**, **B**: Immunofluorescence images of retinal ganglion cell axons traced by CTB-Alexa Fluor 488. The average relative fluorescence intensity in the rIL-4 group was higher than that in the vehicle group on days 7 and 14. **C**, **D**: The cross-sectional area where axons were quantified was 1 cm distal to the injury site. The number of axons labeled with CTB was counted in five areas on a cross section of the optic nerve. Each area was 0.02 × 0.02 (mm^2^). The mean number of CTB-labeled axons in an area was calculated. **E**, **F**: On day 7, the mean axon density labeled by CTB in the rIL-4 group was greater than that in the vehicle group. **G**, **H**: CTB labeling in the lateral geniculate body and superior colliculus of mice. On day 7, the relative fluorescence intensity of CTB in the LGN and SC in the contralateral side of rIL-4 treated eyes was significantly higher than that in the contralateral side of vehicle treated eyes. n = 4 nerves or brains, *** indicates *p* < 0.005, **** indicates *p* < 0.001

We subsequently investigated the neuroprotective effect of interleukin-4 (IL-4) on RGC axons. The structural integrity of CTB-labeled axons in the rIL-4 group was better preserved compared to the vehicle group at both 7 and 14 days post N-methyl-D-aspartate (NMDA) injury. Additionally, there was a significant increase in fluorescence intensity of CTB-labeled axons in the rIL-4 group compared to the vehicle group (Fig. 6A, B). Furthermore, immunofluorescence staining for βIII-tubulin in coronal sections of the optic nerve revealed a significantly higher number of βIII-tubulin-labeled RGC axons in the rIL-4 group on day 7 following NMDA injury (Fig. 6E, F). Moreover, we assessed long-range axon projections from RGCs and observed that on day 7 after NMDA injury, there was a significantly higher fluorescence intensity of CTB labeling in both the lateral geniculate body and superior colliculus within the rIL-4 group compared to the vehicle group (Fig. 6G, H). These findings collectively demonstrate that recombinant IL-4 protein confers protection against NMDA-induced neurotoxicity and preserves long-range axon projection.

**Fig. 7:**
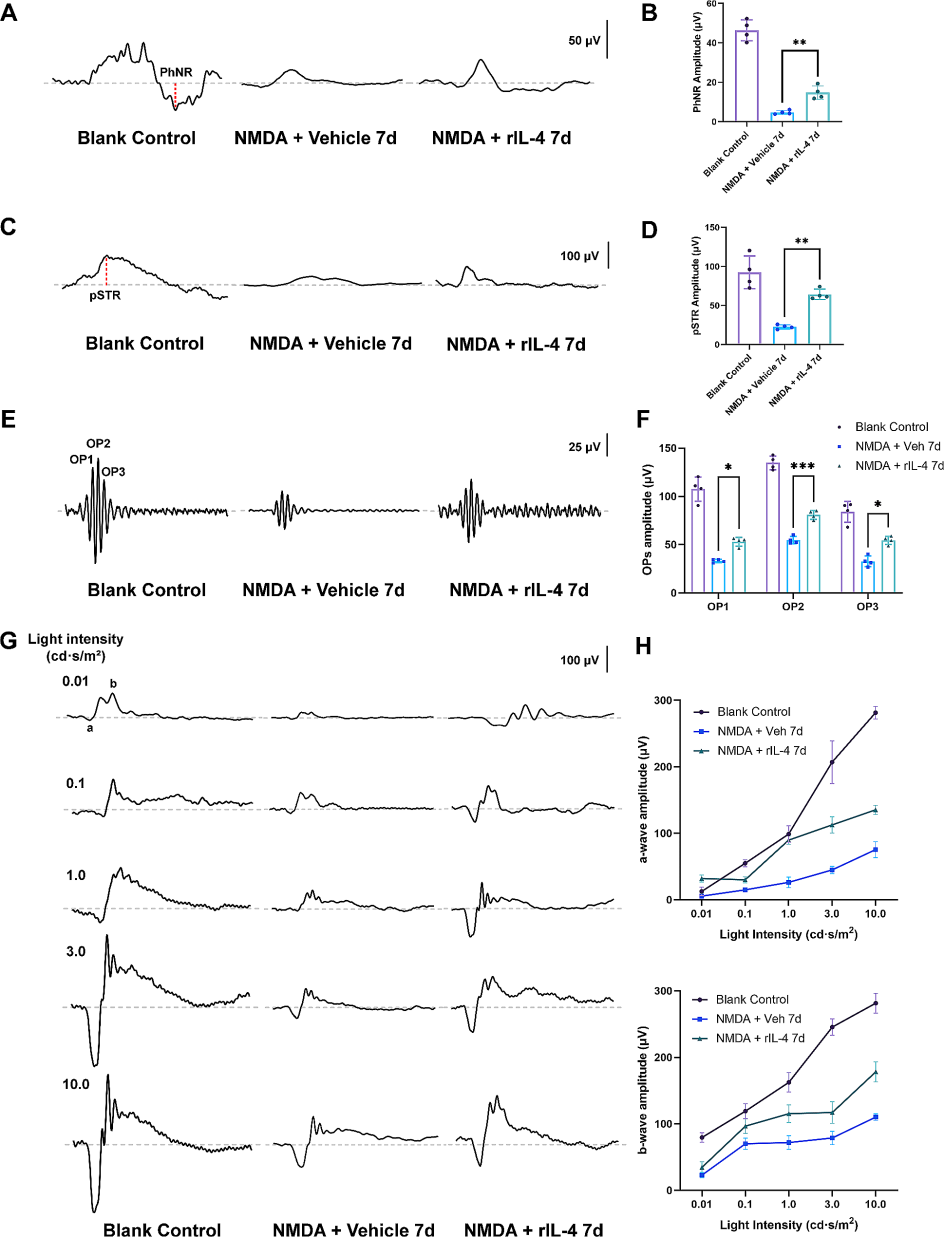
rIL-4 treatment improves ERG performance in mice with NMDA-induced retina injury. **A**: Representative PhNR waveforms from eyes in blank control group, vehicle group and rIL-4 group, after 7-day NMDA damage. The PhNR was elicited with flash stimulus at an intensity of 10.0 cd·s/m^2^ and measured from the baseline to the bottom of the trough following the b wave. **B**: Average amplitudes of PhNR in each group. **C**: Representative pSTR waveforms in each group. The pSTR was elicited with a flash stimulus at an intensity of 0.001 cd·s/m^2^ and measured from the baseline to the following positive peak of the waveform. **D**: Average amplitudes of pSTR in each group. **E**: Representative OP waveforms in each group. The OPs were elicited with the white flashes of 3.0 cd·s/m^2^ and recorded via bandpass filtering between 50 and 170 Hz. **F**: Average amplitudes of OPs (OP1, OP2, and OP3) in each group. **G**: Representative scotopic ERG responses in response to the increasing flash intensity in each group. The flash intensities used to elicit the responses are presented to the left. **H**: Average amplitudes of a- and b- waves in scotopic ERG responses, respectively. n = 4 eyes per group, * indicates *p* < 0.05, ** indicates *p* < 0.01, *** indicates *p* < 0.005

We also conducted flash electroretinogram (ERG) recordings to observe the potential of rIL-4 in improving visual function in the NMDA model. We found that vehicle group exhibited a significant decrease in pSTR amplitude, with a reduction of 69.9 µV at day 7. In contrast, the rIL-4 group experienced a smaller decrease in pSTR amplitude, with a reduction of 28.1 µV. Additionally, the vehicle group displayed a decrease in PhNR (photopic negative response) amplitude by 41.7 µV, while the rIL-4 group showed a smaller decrease of 31.6 µV (Fig. 7A-D). Since pSTR and PhNR originate from RGCs, the reduced amplitudes observed in the vehicle group suggest that the function of RGCs was impaired following NMDA damage. However, rIL-4 was able to mitigate these impairments. Furthermore, rIL-4 treatment also demonstrated an improvement on the amplitude reduction of OPs, a-waves, and b-waves caused by NMDA injury (Fig. 7G, H). These findings indicate that rIL-4 holds the potential to improve visual function in the NMDA-induced retina injury.

### Recombinant IL-4 protein promotes RGC axon regeneration after ONC injury

**Fig. 8:**
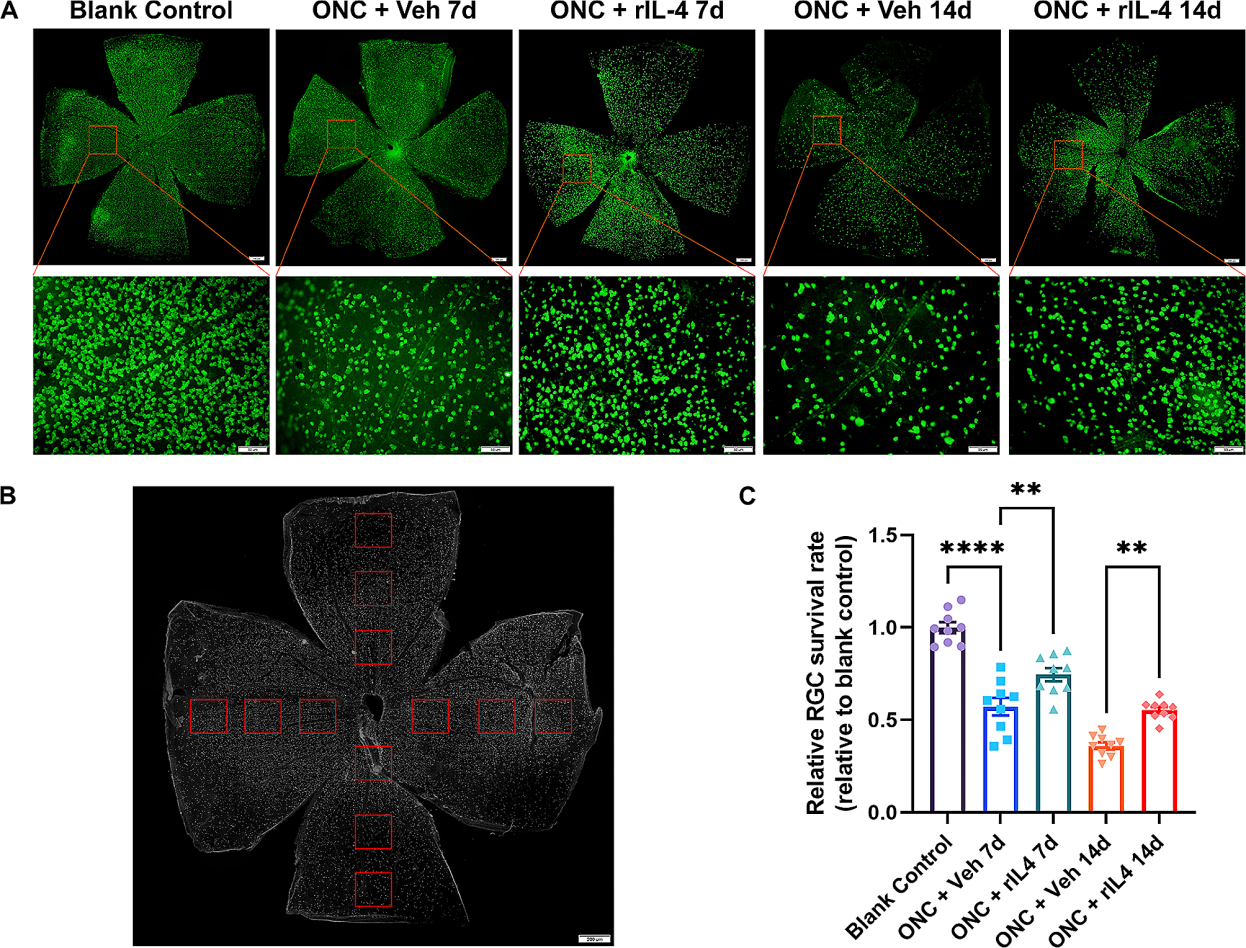
Recombinant IL-4 protein reduced RGC cell death caused by ONC injury. **A**: Immunofluorescence staining of retinal ganglion cells in the negative control group, vehicle group and IL-4 group on day 7 and day 14 showed that the density of RGCs in the rIL-4 group was higher than that in the vehicle group at the same treatment time. **B**: Schematic diagram of the RGC counting method. The calculation method of the relative RGC survival rate was similar to the method of the NMDA model. **C**: Relative RGC survival rate of each group. The relative RGC survival rate of the rIL-4 group was greater than that of the vehicle group with the same treatment time. n = 9 eyes per group, ** indicates *p* < 0.01, **** indicates *p* < 0.001

To assess the efficacy of recombinant IL-4 in the ONC model, we initially evaluated its protective effect on RGCs following ONC injury at first. On days 7 and 14 post-injury, the rIL-4 group exhibited a significantly higher number of surviving RGCs compared to the vehicle group (vehicle group vs. rIL-4 group, day 7: 682 ± 107 vs. 888 ± 69, *p* < 0.05; day 14:585 ± 71 vs. 759 ± 47, *p* < 0.05), indicating that IL-4 confers protection against ONC-induced RGC death (Fig. 8).

**Fig. 9:**
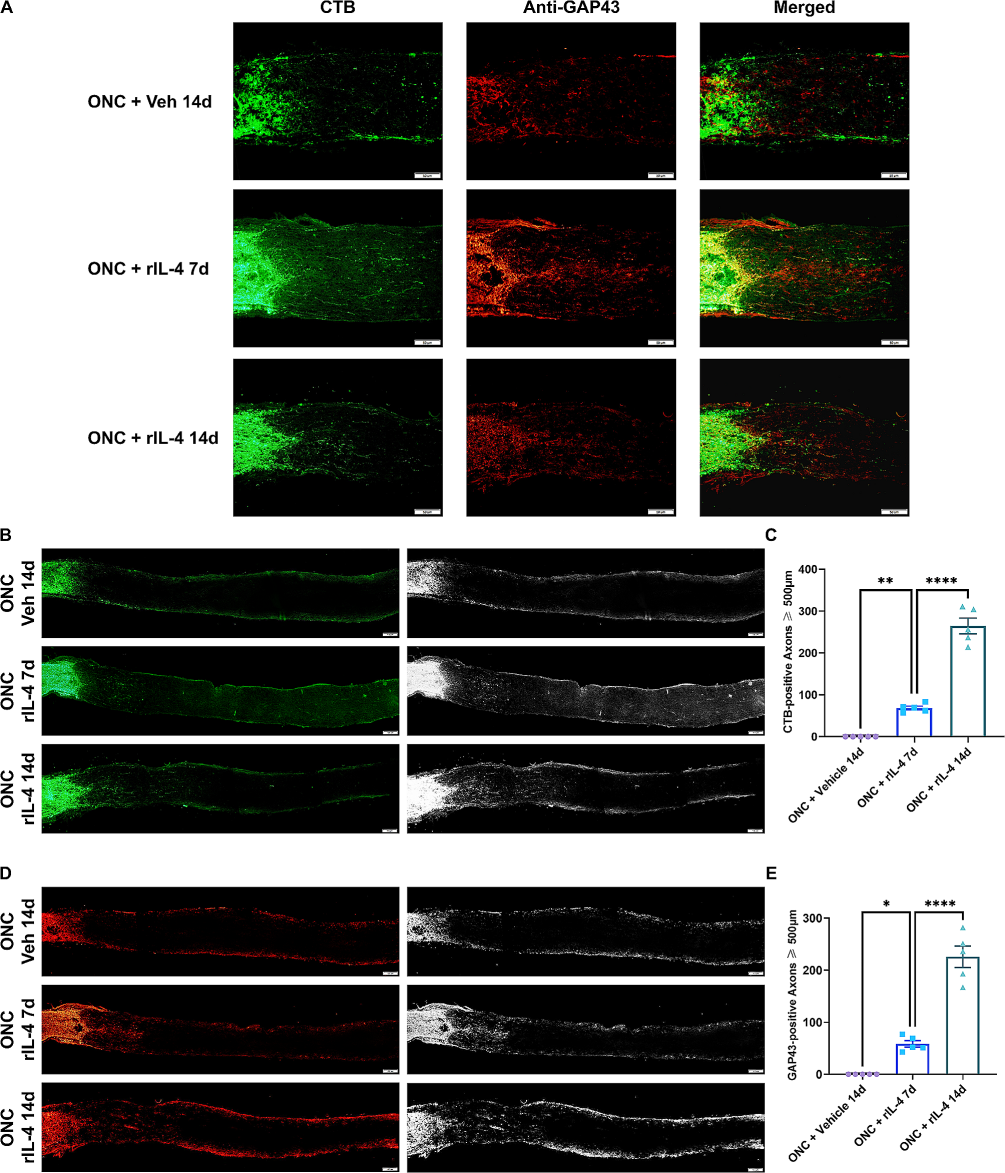
RGC axon regeneration after ONC injury induced by recombinant IL-4 protein. **A**: CTB fluorescence images and GAP43 immunofluorescence staining images of axons distal to the injury site. In the rIL-4 group, there were axons labeled with both CTB (green) and GAP43 (red) distal to the injury site on days 7 and 14. The length of double-labeled axons distal to the injury site was longer in the rIL-4 treatment group on day 14 than on day 7. **B**, **C**: The number of CTB-labeled axons beyond the injury site. The number of regenerated axons with lengths greater than 500 μm beyond the injury site in the rIL-4 group was significantly higher than that on day 7 in the rIL-4 group and that on day 14 in the vehicle group. **D**, **E**: The number of axons beyond the injury site traced by GAP43 immunofluorescence staining. The number of regenerated axons with lengths greater than 500 μm beyond the injury site in the rIL-4 group was significantly higher than that on day 7 in the rIL-4 group and that on day 14 in the vehicle group. n = 5 nerves per group, * indicates *p* < 0.05, ** indicates *p* < 0.01, **** indicates *p <* 0.001

Since growth associated protein-43 (GAP43) is specifically expressed in regenerating axons, we employed CTB antegrade tracing to label RGC axons and performed GAP43 immunofluorescence staining to identify regenerated axons. The axons beyond the site of ONC injury in the rIL-4 group were found to be double-labeled by CTB and GAP43 on days 7 and 14, suggesting their regeneration as evidenced by GAP43 expression (Fig. 9A). Intravitreal administration of rIL-4 significantly enhanced RGC axon regeneration on days 7 and 14 post-injury. The number of regenerated axons extending beyond 500 μm was markedly higher in the rIL-4 group compared to the vehicle group (Fig. 9B, C). Furthermore, the longest regenerated axons reached up to 1.2 mm after ONC injury. Similarly, on days 7 and 14 post-injury, the number of GAP43-positive regenerated axons located more than 500 μm from the injury site was also significantly greater in the IL-4 group compared the vehicle group (Fig. 9D, E). These findings collectively demonstrate that recombinant IL-4 not only protects RGCs from ONC-induced injury but also promotes robust regeneration of their axonal projections.

## Discussion

Staurosporine (STS), an alkaloid isolated from the culture medium of *Streptomyces staurosporesa*, is a broad-spectrum inhibitor of protein kinase C (PKC) [13]. Previous studies have demonstrated that STS induces apoptosis at micromolar concentrations. Interestingly, low concentrations of STS have been shown to significantly promote neurite outgrowth in various neuronal cell types, including HN33 hippocampal cells, RGC-5 retinal cells, PC12 pheochromocytoma cells, and SH-SY5Y neuroblastoma cells [14–16]. These findings suggest that STS may activate a common molecular mechanism underlying axon outgrowth in neurons. In this study, we investigated the key molecular targets involved in STS-mediated axon regeneration using retinal neuronal precursors (661 W cells) [17]. Consistent with previous observations, we observed a significant enhancement of axon outgrowth in 661 W cells treated with low concentrations of STS. To gain insights into the crucial molecular targets responsible for STS-induced axon regeneration, we performed whole transcriptome sequencing (mRNA-Seq) analysis on differentiated 661 W cells. Differential gene expression analysis revealed a significant upregulation of IL-4 gene expression upon treatment with low concentration of STS. Furthermore, protein-protein interaction analysis highlighted IL-4 as a central node regulating multiple inflammation-related signaling pathways.

Subsequently, we confirmed the ability of recombinant IL-4 protein to induce axon outgrowth in 661 W cells. Importantly, exogenous rIL-4 was also found to significantly enhance RGC axon regeneration in the mouse ONC model while providing protection against NMDA-induced retinal excitotoxic injury and preserving long-range projection of RGC axons. Previous studies have demonstrated the neuroprotective role of exogenous IL-4 in central nervous system injury. For instance, He et al. reported that administration of exogenous IL-4 at the site of injury effectively reduced neuronal apoptosis and brain tissue damage caused by cerebral ischemia in mice [18]. Pu et al. on the other hand, utilized nanoparticles for targeted delivery of IL-4 around the injured site in a mouse model of traumatic brain injury, thereby maintaining structural and functional integrity of the brain white matter and promoting transformation of oligodendrocyte precursors into myelin-generating oligodendrocytes [19]. Francos-Quijorn et al. demonstrated that exogenous IL-4 could mitigate inflammation and tissue damage following spinal cord contusions and injury, ultimately improving the spinal cord function [20]. Our findings provide initial evidence supporting an important role for IL-4 as an inflammatory factor involved in RGC axon regeneration and neuroprotection in vivo. Notably, Walsh et al. also observed a similar protective effect exerted by IL-4 on the optic nerve where they identified that CD4^+^ T cells as mediators responsible for protecting RGCs after ONC injury [21].

Previous studies have demonstrated that both NMDA-induced injury and ONC-induced injury can ultimately result in the axon degeneration and death of RGCs [22–24]. NMDA binds to NMDA receptors, leading to an influx of calcium into cells. Calcium overload can trigger RGC death through various mechanisms, including activation of apoptotic pathways, calcium-dependent proteases activation, and generation of free radicals [25]. Following ONC injury, the axon structure is compromised, leading to axonal degeneration and retrograde RGC death due to excessive calcium influx, inflammation, oxidative stress, and other mechanisms. IL-4 has been shown to inhibit the activation of apoptosis pathway and reduce inflammation [18, 26]. Furthermore, Zhou et al. demonstrated that exogenous IL-4 could enhance the secretion of brain-derived neurotrophic factor (BDNF), ciliary neurotrophic factor (CNTF) and insulin-like growth factor-1 (IGF-1) in a mouse model of ischemic brain injury [27]. BDNF, CNTF and IGF-1 have been reported to attenuate NMDA-induced neuronal cell death [28–30]. Therefore, IL-4 may exert its protective effect on RGCs through diverse mechanisms.

On day 7 post-ONC injury, the axonal structure of RGCs distal to the injury site disintegrated in both the negative control group and the vehicle group. In contrast, regenerated RGC axons were observed in the rIL-4 group, which exhibited expression of GAP43 protein and could be traced by CTB, indicating their functional axon transport capability. Notably, our findings demonstrated that recombinant IL-4 protein exerts a prolonged effect on RGC axon regeneration lasting until at least day 14, contrary to previous studies [31]. The mechanism underlying IL-4-mediated promotion of axon regeneration is intricate. Apart from its direct action on mouse RGCs, IL-4 can induce immune cells to secrete nerve growth factors such as TGF-β2 and NGF that facilitate nerve regeneration through diverse mechanisms [32, 33]. Additionally, Golz et al. demonstrated that IL-4 promotes axon regeneration in mouse DRG cells when combined with neurotrophic factors like neurotrophin-4 (NT-4) [34]. Furthermore, inflammation-related factors contribute to neuronal cell death. For instance, Liddelow et al. reported that inflammation-induced activation of A1 astrocytes leads to cell death and axonal degeneration following ONC injury in RGCs [35]. However, a study conducted on spinal cord tissue have revealed that glial scar formation restricts short-term diffusion of inflammation at the injury site while impeding long-term axon regeneration [36]. Inflammatory factors may facilitate axon regeneration by attenuating glial scarring through promoting phagocytosis of debris, clearance of debris, and sprouting of axonal protrusions [37–40]. Furthermore, following central nervous system (CNS) injury, polarization of microglia and infiltrating macrophages towards an M2 phenotype with enhanced phagocytic capacity promotes myelin debris removal and reduces scar formation, thereby establishing a conducive microenvironment for axon regeneration [41]. Liu et al. demonstrated that IL-4 plays a pivotal role in facilitating M2 polarization of microglia/macrophages, thus contributing to long-term recovery after cerebral ischemic injury [42]. Additionally, Park et al. revealed that IL-4 and IL-10 can promote axon regeneration in sensory neurons by mitigating inflammation and modulating local immune responses following spinal cord injury in mice [43]. In summary, IL-4 may exert its pro-regenerative effects on CNS injuries through diverse mechanisms.

Additionally, IL-4 induces the phosphorylation of STAT6 and ERK. Previous studies have demonstrated that activation of the IL-4 receptor can mediate the activation of the STAT6 signaling pathway. The phosphorylated STAT6 translocates to the nucleus where it regulates transcription of IL-4-responsive genes, ultimately leading to recruitment of the MAPK signaling cascade [44]. The recruitment of MAPK signaling, including ERK, has been shown to positively contribute to axon regeneration following injury in both central and peripheral nervous systems [45–47]. Deboy et al. provided evidence that CD4^+^ T cells exert a protective effect on motor neurons in facial nerve transection mice through dependence on the IL-4/STAT6 signaling pathway. This protective effect was significantly weakened in IL-4 knockout mice [48]. Therefore, recombinant IL-4 protein may enhance axon regeneration in retinal ganglion cells after optic nerve crush by activating both JAK1/STAT6 and ERK signaling pathways.

## Conclusion

The present study provides the initial evidence that exogenous recombinant IL-4 confers protective effects on mouse retinal ganglion cells (RGCs) against injuries induced by N-methyl-D-aspartate (NMDA) and optic nerve crush (ONC). Additionally, IL-4 preserves the integrity of long-distance RGC axon projections following NMDA injury and enhances axon regeneration in RGCs after ONC injury. Consequently, IL-4 emerges as a promising molecular target for therapeutic interventions aimed at mitigating disorders associated with RGC degeneration, such as traumatic optic neuropathy and glaucoma.

### Electronic supplementary material

Below is the link to the electronic supplementary material.

Supplementary Material 1

## Data Availability

No datasets were generated or analysed during the current study.

## Abbreviations

AKT: protein kinase B
BDNF: brain-derived neurotrophic factor
BSA: bovine serum albumin
CCD: charge-coupled device
CNS: central nerve system
CNTF: ciliary neurotrophic factor
CTB: cholera toxin subunit B
DAPI: 4’,6-diamidino-2-phenylindole
ERK: extracellular regulated protein kinases
GAP43: growth associated protein-43
IGF-1: insulin-like growth factor-1
IL-4: interleukin-4
IL-6: interleukin-6
IL-10: interleukin-10
JAK: Janus kinase
KEGG: Kyoto Encyclopedia of Genes and Genomes
MAPK: mitogen-activated protein kinase
mTOR: mammalian target of rapamycin
NGF: nerve growth factor
NMDA: N-methyl-D-aspartic acid
NMDAR: N-methyl-D-aspartic acid receptor
ONC: optic nerve crush
PBS: phosphate buffered saline
PCR: polymerase chain reaction
PI3K: phosphatidylinositol 3 kinase
PKC: protein kinase C
PTEN: phosphatidylinositol-3,4,5-trisphosphate 3-phosphatase and dual-specificity protein phosphatase
RBPMS: RNA binding protein, mRNA processing factor
RGC: retinal ganglion cell
rIL-4: recombinant IL-4 protein
STAT3: Signal Transducer and Activator of Transcription 3
STS: staurosporine
TGF-β2: transforming growth factor-β2
TNF-α: tumor necrosis factor-α
