# Supplementary material for: Interleukin-4 protects retinal ganglion cells and promotes axon regeneration
Source: Cell Commun Signal. 2024 Apr 22;22:236. doi: 10.1186/s12964-024-01604-y (PMC11034112; doi:10.1186/s12964-024-01604-y)
Supplement: Supplementary file 1 — Supplementary Material 1 [file 12964_2024_1604_MOESM1_ESM.docx]

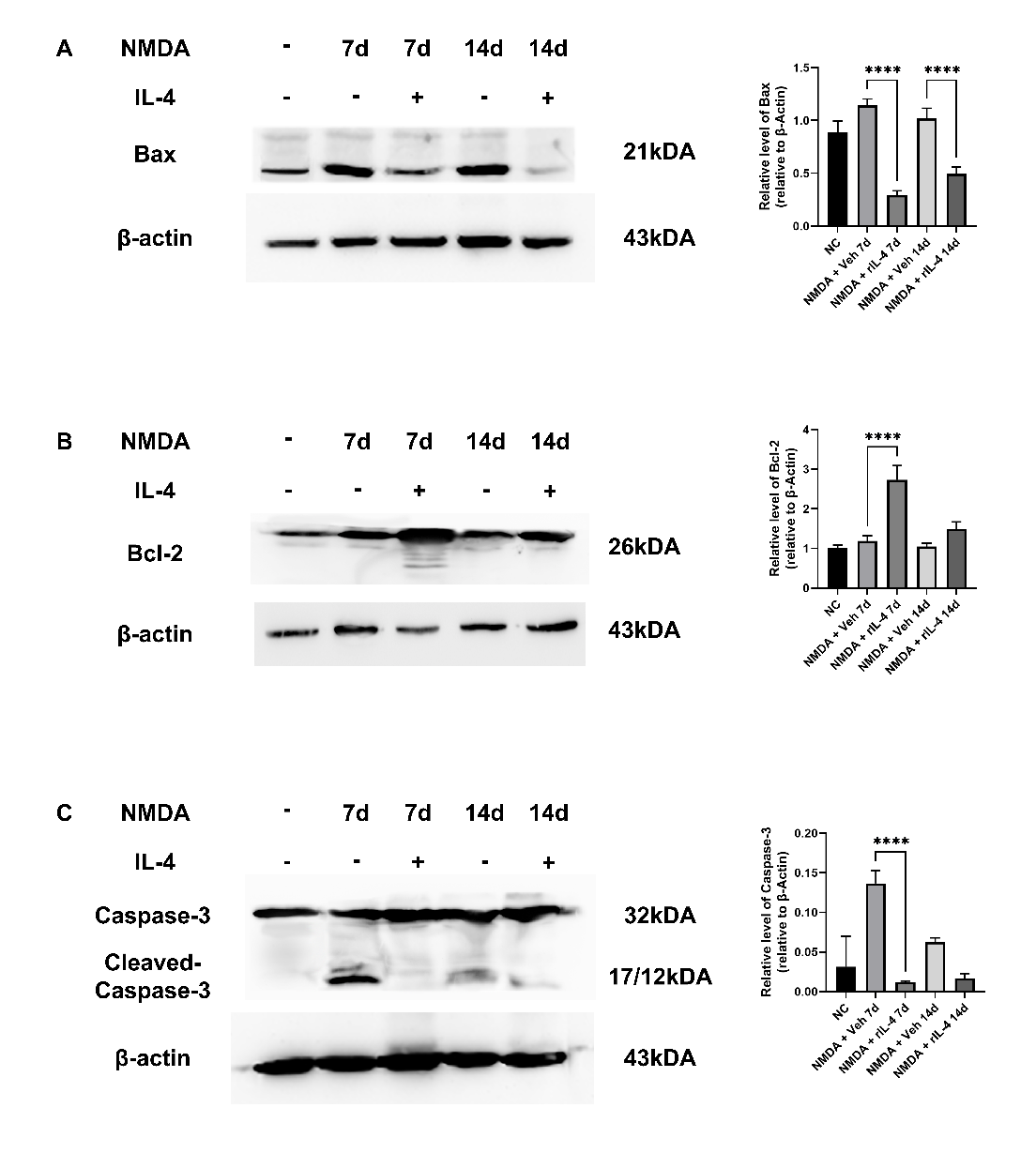
 Figure S1. Western blot analysis revealed that the administration of exogenous recombinant IL-4 protein (rIL-4) significantly upregulated the expression of Bcl-2, while concurrently suppressing the levels of BAX and Caspase-3 in the retina of the NMDA-induced injury model.
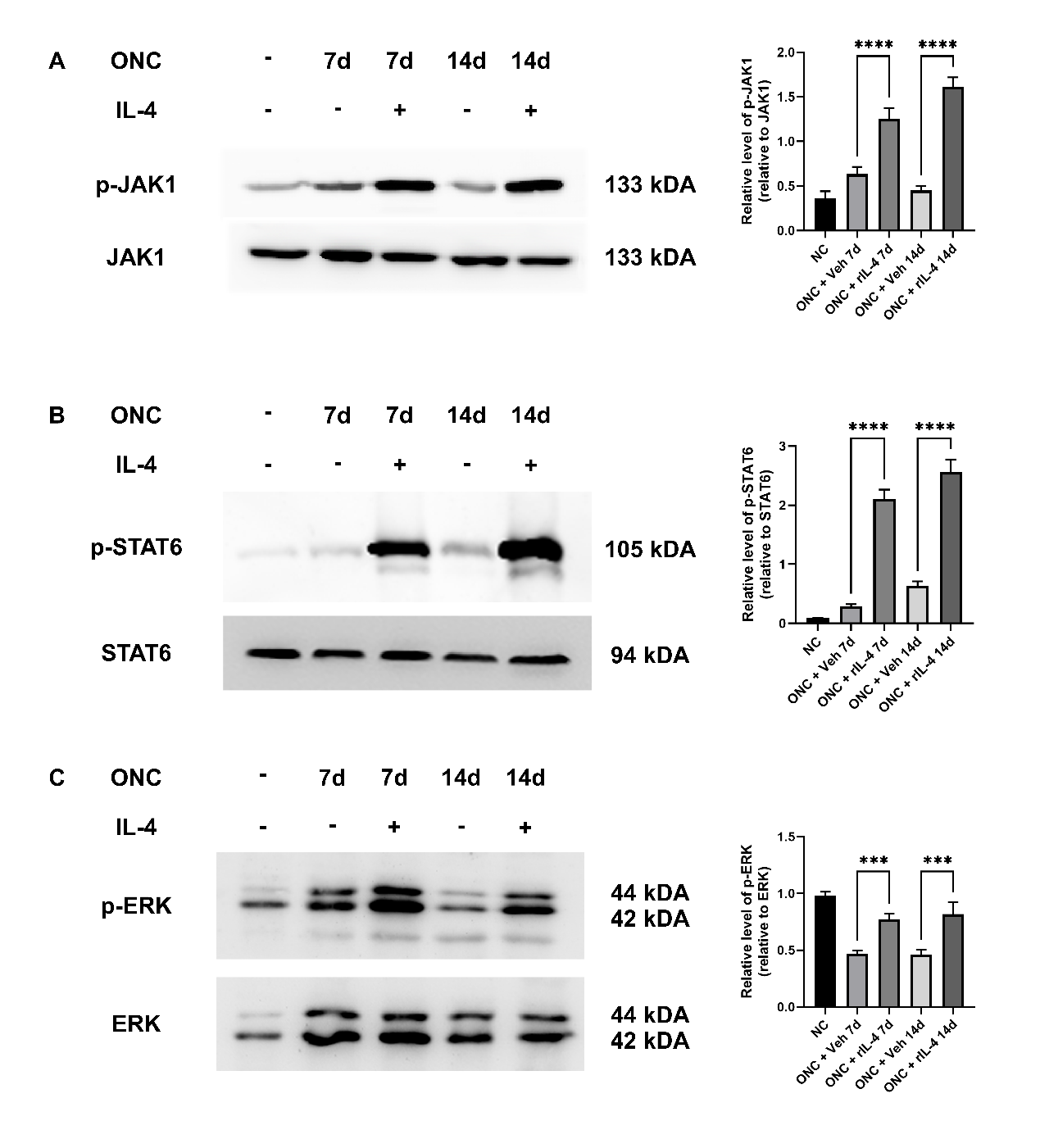


Figure S2. Western blot analysis indicated that rIL-4 treatment promoted the phosphorylation of JAK1/STAT3 and ERK1/2 in the retina of the ONC model, resulting in the activation of the associated signaling cascades.

**Table 1. Chemicals and reagents used in the study**

| **Chemicals** | **Source** | **Identifier** | **Dosage** |
| --- | --- | --- | --- |
| Staurosporine | Abmole (Beijing, China) | Cat# M2066 |  |
| N-methyl-D-aspartic Acid (NMDA) | Abmole | Cat# M2884 |  |
| 4’,6-diamidino-2-phenylindole (DAPI) | Solarbio (Beijing, China) | Cat# C0065 |  |
| Antifading mounting medium | Solarbio | Cat# S2100 |  |
| Cholera Toxin Subunit B (CTB) conjugated Alexa Fluor 488 | Thermo Fisher Scientific (MA, USA) | Cat# C34775 |  |
| recombinant murine IL-4 protein | PeproTech (Rocky Hill, NJ, USA) | Cat# 214-14 |  |
| **Reagents for cell histology** | **Source** | **Identifier** | **Dosage** |
| mouse anti-βIII-Tubulin | Beyotime (Shanghai, China) | Cat# AT809 | 1:100 |
| rabbit anti-NeuN | Abcam (Cambridge, UK) | Cat# ab177487 | 1:400 |
| DyLight 488, anti-mouse IgG | Abbkine (Shanghai, China) | Cat# A23210 | 1:400 |
| Cy3, anti-rabbit IgG | Abbkine | Cat# A22220 | 1:400 |
| **Reagents for Western blot** | **Source** | **Identifier** | **Dosage** |
| mouse anti-β-III Tubulin | Beyotime | Cat# AT809 | 1:2000 |
| rabbit anti-NeuN | Abcam | Cat# ab177487 | 1:5000 |
| mouse anti-β-actin | Signalway (Pearland, TX, USA) | Cat# 21800 | 1:8000 |
| anti-rabbit IgG, HRP conjugated | Signalway | Cat# L3012 | 1:8000 |
| anti-mouse IgG, HRP conjugated | Signalway | Cat# L3032 | 1:8000 |
| Reagents for tissue histology | Source | Identifier | Dosage |
| rabbit anti-RBPMS | OmnimAbs (California, USA) | Cat# OM165217 | 1:200 |
| rabbit anti-GAP43 | Beyotime | Cat# AF0153 | 1:200 |
| mouse anti-β-III Tubulin | Beyotime | Cat# AT809 | 1:100 |
| rabbit anti-IL4R | Affinity Biosciences | Cat# DF8567 | 1:200 |
| DyLight 488, anti-rabbit IgG | Abbkine | Cat# A23220 | 1:400 |
| DyLight 488, anti-mouse IgG | Abbkine | Cat# A23210 | 1:400 |
| Cy3, anti-rabbit IgG | Abbkine | Cat# A22220 | 1:400 |
